# Assessment of in vitro anti-skin aging activities of *Phyllanthus indofischeri* Bennet extracts for dermatological and aesthetic applications

**DOI:** 10.1038/s41598-023-45434-3

**Published:** 2023-10-31

**Authors:** Korawinwich Boonpisuttinant, Thanachai Taka, Warintorn Ruksiriwanich, Romchat Chutoprapat, Sarinporn Udompong, Rattiya Kansawang, Jinapa Sangsee, Wirinda Chompoo, Kitrawi Samothai, Ratakorn Srisuttee

**Affiliations:** 1 Innovative Natural Products from Thai Wisdom Research Unit, Faculty of Integrative Medicine, Rajamangala University of Technology Thanyaburi, Pathumthani, 12130 Thailand; 2 iCell Research Institute Laboratory Unit, Bangkok, 10230 Thailand; 3 Department of Pharmaceutical Sciences, Faculty of Pharmacy, Chiang Mai University, Chiang Mai, 50200 Thailand; 4 Center of Excellence in Agro Bio-Circular-Green Industry (Agro BCG), Agro-Industry, Chiang Mai University, Chiang Mai, 50100 Thailand; 5 Lanna Rice Research Center, Chiang Mai University, Chiang Mai, 50200 Thailand; 6 Department of Pharmaceutics and Industrial Pharmacy, Faculty of Pharmaceutical Sciences, Chulalongkorn University, Bangkok, 10330 Thailand; 7 Faculty of Medicine, King Mongkut’s Institute of Technology Ladkrabang, Bangkok, 10520 Thailand

**Keywords:** Chemical biology, Molecular biology, Plant sciences, Molecular medicine

## Abstract

Giant Indian Gooseberry (GIG) or *Phyllanthus indofischeri* Bennet are commercially cultivated and commonly used herbs in Traditional medicine, especially in Thailand. The aim of this study was to assess the potential of the GIG extracts in anti-aging activities to be a dermatological application. The juice, meat residues, and seeds of GIG collected from Sra Kaeo Province, Thailand, were extracted by the Boiling method (B) and the Maceration process (M) by using 95% ethanol as a solvent. The GIG extracts gave the total phenolic, total flavonoid contents and quercetin contents, as well as exhibited anti-oxidative activities. The GIG-R-B extract inhibited tyrosinase activity and had the highest anti-melanogenesis activity on B_16_F_10_ cells at 31.63 ± 0.70%. The GIG-S-B, GIG-S-M, and GIG-R-M extracts demonstrated the highest collagen biosynthesis, which was comparable to vitamin C (*p* < *0.05*), whereas the GIG-R-B extracts gave the highest stimulation of anti-aging genes (*SIRT1* and *FOXO1*). All extracts at the concentration of 0.1 mg/mL showed no cytotoxicity on human skin fibroblasts. Therefore, the GIG-S-B extract was discovered to be a promising natural anti-aging agent for dermatological health and aesthetic applications that can be further developed in cosmetic, functional food and food supplement industries.

## Introduction

The skin is the largest organ that covers the entire human body. A good skin appearance is relevant to social activity by improving self-confidence and making a favorable impression. However, chronologically aged skin, such as wrinkles, xerosis, laxity, and slackness, as well as melanin overproduction, such as melasma, freckles, dark spots, lentigo and hyperpigmentation disorders, can be aesthetically undesirable during the aging process^1^^,^^2^. Skin aging is affected by exogenous factors such as Ultraviolet radiation, xenobiotics, air pollutants, and smoking and by endogenous factors such as cellular metabolism, hormones, metabolic processes, and genetics^3^. The reactive oxygen species (ROS) from both exogenous and endogenous are responsible for the activation of inflammatory processes and signaling pathways on skin aging, leading to the inducing of apoptotic cell death, increasing of metalloproteinases (MMPs) levels that affect the structures and levels of collagens and elastin^3,4^, as well as the regulation of oxidative stress in melanocytes resulting to skin hyperpigmentation^2^. SIRT1 is a nuclear NAD+ -dependent class III histone deacetylase, the most well-studied to have anti-aging effects in both animals and humans^5^. SIRT1 regulates apoptosis, cell senescence, and cell growth, involving regulation of metabolism, cell survival, differentiation, and aging^5^. SIRT1 exerts its anti-aging effects by deacetylating FOXO1, a transcription factor of the family members in forkhead box protein found in the cytoplasm and activated through mitogen-activated protein kinase (MAPK), protein kinase (AKT), and pancreatic and duodenal homeobox-1 (Pdx1) pathway^6^. The increasing of *SIRT1* and *FOXO1* mRNA expression regulates DNA repair, neuroprotection, and vascular protection and decreases aging and age-related diseases such as cellular senescence, oxidative stress, inflammation, neurodegeneration, cardiovascular diseases, adiposity, insulin resistance and liver steatosis as well as skin aging^5,7^.

The use of herbs and natural extracts for skin aging has a long history dating back thousands of years. Natural anti-skin aging agents or extracts from natural sources have been derived from minerals, animals; and vegetables and herbs, which have been several reported for dermatological health and aesthetic applications including cosmetics, cosmeceuticals and pharmaceuticals^8^. For example, the extracts from *Hypoxis aurea* Lour., *Scabiosa columbaria* L., and *Vigna subterranean* has been shown to have anti-oxidative, anti-melanogenic, and mushroom tyrosinase inhibitory effects, as well as collagen biosynthesis-promoting properties^9–11^. Resveratrol, which is derived from grape, apigenin, and luteolin, has also been shown to activate the expression of SIRT1 and FOXO1 mRNA and proteins^12,13^ that may help to protect the skin from damage and aging.

Indian Gooseberry, popularly known as amla, is from two species, *Phyllanthus emblica* L. and *Phyllanthus indofischeri* Bennet, that are commercially cultivated and commonly used herbs in Indian Ayurvedic systems^14^. These two species are classified by their morphological such as bark color, leaf and fruit color, and fruit sizes; and reproductive parameters such as number of male and female flowers, fruit set and retention, the fruits, and rate of fruit maturity^14^. The bark color, branchlet length, fruit size (length and width), and grooves of *P. emblica* L. are brown, 38–40 cm, and 1.8 and 2.5 cm, respectively, whereas gray, 20–25 cm, and 2.5 and 4.0 cm are found in *P. indofischeri* Bennet^14^. Giant Indian Gooseberry (*P. indofischeri* Bennet) has a rich of vitamin C, and presences of alkaloids, ellagitannins, gallic acid, emblicanin A and emblicanin B, flavonoids (especially rutin and quercetin)^14^. Although, it has several reports for biological activity of *P. emblica* L. such as chemo-preventive, anti-diabetic, anti-microbial, anti-inflammatory, analgesic, anti-mutagenic, antioxidant, diuretic, aphrodisiac, UV protectant, and anti-aging activities^15^, but there are a few scientific reports for *P. indofischeri* Bennet especially anti-aging activity for dermatological health and aesthetic purposes. Herein, the aim of this study was to assess the potential of the Indian Gooseberry extracts from *P. indofischeri* Bennet specie to be a dermatological application. The in vitro anti-aging activity of the Giant Indian Gooseberry extracts including activation of *SIRT1* and *FOXO1* mRNA expression and collagen biosynthesis on human dermal fibroblasts, tyrosinase inhibition and anti-melanogenesis on murine melanomas (B_16_F_10_), as well as anti-oxidative activities including the free radical scavenging, lipid peroxidation inhibition, and metal chelation activity were investigated.

## Methods

### Preparation and extraction

The fruits of Giant Indian Gooseberry (GIG) were collected in Sra Kaeo Province, Thailand, from August to December 2020. The GPS coordinates of the collection site are 13.8077° N, 102.0917° E. The collection was done in accordance with Thailand’s guidelines and legislation. The voucher specimens (RSPG-RMUTT-101) have been authenticated by a botanist and are kept at the Faculty of Integrative Medicine, Rajamangala University of Technology Thanyaburi (RMUTT), Pathum Thani, Thailand. The preparation and extraction of the GIG extract were done following the previous methods with some modifications^16^. The GIG fruits were washed with tap water and halved with a sharp knife to remove their seeds. The meats were squeezed by a squeezer to extract the raw juice, which was then filtered and lyophilized to obtain the lyophilized juice (GIG-J-L). The meat residues and seeds were dried at 60 °C in a hot air oven, ground into powder, and extracted using two methods: maceration with 1 L of 95% ethanol for 48 h (GIG-R-M and GIG-S-M), or boiling with 100 mL of distilled water for 2 h (GIG-R-B and GIG-S-B). The extracts were filtered and concentrated using a rotary evaporator at the temperature of 50 °C. The crude extracts were stored in glass bottles at 4 °C until use. The extraction yields were calculated based on dry weight.

### Phytochemical analysis, total phenolic, total flavonoid and quercetin contents

Phytochemical constituents such as alkaloids, flavonoids, glycosides, saponin, tannins, terpenoids, and xanthones of the samples were investigated as previously described^17^. The qualitative results are expressed as (+) for the presence and (−) for the absence of phytochemicals. A sample solution was prepared by dissolving 20 mg of the extracts in 20 ml of distilled water, and then phytochemical tests were performed. *Alkaloids*: 2 ml of the extract solution was mixed with 1 ml of 1% HCl and boiled on a water bath. 6 drops of Dragendorff's reagent were added. A creamy, brownish-red, or orange precipitate indicated the presence of alkaloids. *Flavonoids*: 2 ml of the extract solution was mixed with 1 ml of concentrated HCl and magnesium ribbon. A pink or tomato-red color indicated the presence of flavonoids. *Glycosides* (Fehling's test for reducing sugars): 2 ml of the extract solution was mixed with 1 ml of Fehling's solution and heated in a water bath for 10 min. A brick-red precipitate indicated the presence of reducing sugar in glycosides. *Saponins*: 2 ml of the extract solution was shaken with distilled water (10 ml) in a test tube. The formation of frothing, which persisted on warming in a water bath for 5 min, indicated the presence of saponins. *Tannins*: 2 ml of the extract solution was mixed with 2 ml of 15% FeCl_3_ solution. A blue-black precipitate indicated the presence of tannins. *Terpenoids*: 2 ml of the extract solution was shaken with chloroform (2 ml) followed by the addition of concentrated H_2_SO_4_ (2 ml) along the side of the test tube. A reddish-brown coloration of the interface indicated the presence of terpenoids. *Xanthones*: 2 ml of the extract solution was mixed with 1 ml of 5% KOH reagent. The formation of a yellow precipitate indicated the presence of xanthones.

The total phenolic content (TPC) of the GIG extracts was determined using the Folin-Ciocalteu assay, as previously described^9^. Briefly, 50 µL of each extract was added to a 96-well microplate, followed by 75 µL of Folin-Ciocalteu reagent (Global Chemie, Mumbai, Maharashtra, India) and 75 µL of 7.5% Na_2_CO_3_. The plates were then incubated at room temperature in the dark for 90 min. The absorbance of the resulting solutions was measured at 580 nm using a microplate reader (VICTOR^®^ Nivo, PerkinElmer, USA). The TPC of each sample was calculated from a standard curve of gallic acid (Sigma Aldrich-Merck KGaA, Darmstadt, Germany).

The total flavonoid contents (TFC) of the GIG extracts were determined using the modified aluminum chloride method, as previous described^18^. Briefly, 50 µL of each extract was added to a 96-well microplate, followed by 25 µL of 10% AlCl3 (VWR Chemicals BDH, Radnor, Pennsylvania, USA.) and 100 µL of 5% NaNO2 (Global Chemie, Mumbai, Maharashtra, India). After 5 min of incubation, 25 µL of 1 M NaOH (VWR Chemicals BDH, Radnor, Pennsylvania, USA.) was added and the reaction was allowed to proceed at room temperature for 10 min. The absorbance of the resulting solutions was measured at 420 nm using a microplate reader. The TFC of each sample was calculated from a standard curve of quercetin. The results were expressed as milligrams of quercetin equivalents (QE) per gram of extract.

The quercetin contents of the GIG extracts were determined using high-performance liquid chromatography (HPLC)^19^. Briefly, a standard quercetin solution was prepared by dissolving 1000 µg of quercetin in methanol in a 100 mL volumetric flask. The stock solution was then serially diluted to obtain a range of concentrations. For sample preparation, the extract samples were dissolved in methanol (Fisher Scientific, Loughborough, UK) to obtain a concentration of 1000 mg/mL. All solutions and solvents were filtered through a 0.22 µm filter and degassed. The HPLC analysis was performed using an Agilent 1260 infinity HPLC system (Waters, Millford, MA, USA) equipped with a photodiode array detector. The analytical column was an Agilent Zorbax extended column C18 with dimensions of 5 μm × 100 mm × 4.6 mm. The mobile phase was methanol: 0.1% ortho phosphoric acid (Merck, Darmstadt, Germany) (75:25), and the flow rate was 0.6 mL/min. The sample injection volume was 10 µL, and the run time was 8 min. The column temperature was maintained at 25 °C, and the detection wavelength was set at 370 nm.

### Anti-oxidative activities

#### Free radical scavenging activity by 2,2-diphenyl-1-picrylhydrazyl (DPPH) method

The free radical scavenging activity of the samples was determined using the modified DPPH method, as described in (9, 17). Briefly, 100 µL of each extract and 100 µL of 0.1 mg/mL of DPPH (Sigma Aldrich-Merck KGaA, Darmstadt, Germany) solution in absolute ethanol (Merck, Darmstadt, Germany) were added to a 96-well microplate. After incubation at room temperature in the dark for 30 min, the absorbance of the resulting solutions was measured at 515 nm using a microplate reader. Vitamin C (L-ascorbic acid) (Sigma Aldrich-Merck KGaA, Darmstadt, Germany) was used as the positive control. The experiments were repeated in triplicate. The percentages of free radical scavenging activity were calculated using the following formula:

$$\% \,{\text{Free}}\,{\text{radical}}\,{\text{scavenging}}\,{\text{activity}} = \left[ {{\text{A}}_{0} - {\text{A}}_{{1}} /{\text{A}}_{0} } \right] \times {1}00$$

where A_0_ is the absorbance of the control and A_1_ is the absorbance of the samples. The concentrations providing 50% scavenging (SC_50_) were then extrapolated from the graph plotted between the %free radical scavenging and the sample concentrations.

#### Lipid peroxidation inhibition by the modified Ferric-thiocyanate method

The lipid peroxidation inhibition of the samples was determined using the modified Ferric-thiocyanate method^9,17^. Briefly, 50 µL of each extract and 50 µL of linoleic acid (Sigma Aldrich-Merck KGaA, Darmstadt, Germany) in 50% DMSO (SRL, Maharashtra, India) were added to a 96-well microplate. The reaction was initiated by the addition of 50 µL of 5 mM NH_4_SCN (Global Chemie, Mumbai, Maharashtra, India) and 50 µL of 2 mM FeCl_2_ (Sigma Aldrich-Merck KGaA, Darmstadt, Germany). After incubation at 37 °C in the dark for 60 min, the absorbance of the resulting solutions was measured at 490 nm using a microplate reader. Vitamin E (α-tocopherol) (Sigma Aldrich-Merck KGaA, Darmstadt, Germany) was used as the positive control. The experiments were repeated in triplicate. The percentages of lipid peroxidation inhibition were calculated using the following formula:

$$\% \,{\text{Lipid}}\,{\text{peroxidation}}\,{\text{inhibition}} = \left[ {\left( {{\text{A}}_{0} - {\text{A}}_{{1}} } \right)/{\text{A}}_{0} } \right] \times {1}00$$

The concentrations providing 50% of lipid peroxidation inhibition (LC_50_) were extrapolated from the graph plotted between the % lipid peroxidation inhibition and the sample concentrations.

#### Metal chelation by FIC method

The metal chelation of the samples was determined using the FIC method^9,17^. Briefly, 50 µL of each extract, 1 mg/mL FeCl2, and 50 µL of 1 mg/mL ferrozine (TCI, Chuoku, Tokyo, Japan) in 1% HCl (Sigma Aldrich-Merck KGaA, Darmstadt, Germany) were added to a 96-well microplate. The microplate was incubated at room temperature in a dark place for 60 min, and then the absorbances at the wavelength of 570 nm were measured using a microplate reader. EDTA (ethylenediaminetetraacetic acid) (Global Chemie, Mumbai, Maharashtra, India) was used as the positive control. The percentages of metal chelation were calculated using the following formula:

$$\% \,{\text{Metal}}\,{\text{chelation}} = \left[ {\left( {{\text{A}}_{0} - {\text{A}}_{{1}} } \right)/{\text{A}}_{0} } \right] \, \times { 1}00$$

The concentrations providing 50% of metal chelation (MC_50_) were then extrapolated from the graph plotted between the % metal chelation and the sample concentrations.

### Mushroom Tyrosinase inhibition activity

The mushroom tyrosinase inhibition activity of the samples was assayed by the modified dopachrome method using tyrosine as a substrate^9,17^. Briefly, 50 µL of the samples, 50 µL of 0.1 mg/mL L-tyrosine (Sigma Aldrich-Merck KGaA, Darmstadt, Germany), 50 µL of 0.1 mg/mL mushroom tyrosinase (Sigma Aldrich-Merck KGaA, Darmstadt, Germany), and 50 µL of 0.1 mM phosphate buffer were added to a 96-well microplate. The microplate was incubated at room temperature in a dark place for 60 min, and then the absorbances at the wavelength of 570 nm were measured using a microplate reader. Kojic acid (SRL, Maharashtra, India) was used as the positive control. The percentages of mushroom tyrosinase inhibition were calculated using the following formula:

$$\% \,{\text{Mushroom}}\,{\text{tyrosinase}}\,{\text{inhibition}} = \left[ {\left( {{\text{A}} - {\text{B}}} \right) - \left( {{\text{C}} - {\text{D}}} \right)} \right]/\left( {{\text{A}} - {\text{B}}} \right) \times {1}00$$

where A is the absorbance of the blank after incubation, B is the absorbance of the blank before incubation, C is the absorbance of the samples after incubation, and D is the absorbance of the samples before incubation. The concentrations providing 50% tyrosinase inhibition (IC_50_ mg/mL) were then extrapolated from the graph plotted between % tyrosinase inhibition and the sample concentrations.

### Cell cultures

The dermal human skin fibroblasts (ATCC CRL-2932) were obtained from the American Type Culture Collection (ATCC) in Virginia, USA. These cells were used to investigate the cytotoxicity, stimulation of collagen biosynthesis, and anti-aging *SIRT1* and *FOXO1* mRNA expression. The murine melanomas (B_16_F_10_) (ATCC CRL-6166) were also obtained from ATCC. These cells were used to investigate the cytotoxicity and anti-melanogenesis. Both cell lines were cultured in the Dulbecco's Modified Eagle Medium (DMEM) (Gibco-Invitrogen, Waltham, Massachusetts, USA) supplemented with 10% fetal bovine serum (FBS) (Gibco-Invitrogen, Waltham, Massachusetts, USA), 100 IU/ml of penicillin and streptomycin (Gibco-Invitrogen, Waltham, Massachusetts, USA) under the standard conditions (37 °C and 5% CO_2_ atmosphere) before the experiments.

### Cytotoxicity test by MTT assay

The cytotoxicity of the samples at various concentrations on human skin fibroblasts was assayed using the 3-(4,5-dimethylthiazol-2-yl)-2,5-diphenyltetrazolium bromide (MTT) method, as described previously^9,17^. A density of 1 × 10^4^ cells of human skin fibroblasts were seeded into a sterile 96-well plate and adjusted the volume to 180 µL with DMEM medium. The cells were incubated at the standard conditions for 24 h. After incubation, the cells were treated with 20 µL of the samples and then incubated at the standard conditions overnight. Subsequently, the medium was removed and the cells were gently washed with 10 mM phosphate buffer saline (PBS) at pH 6.8 for 3 times. An amount of 200 µL of MTT (Sigma Aldrich-Merck KGaA, Darmstadt, Germany) solution at 0.5 mg/mL was added to each well and further incubated at the standard conditions for 3 h. Then, the MTT solution was removed and 100 µL of dimethyl sulfoxide (DMSO) was added to the plates to dissolve the blue-violet crystals. The plates were gently shaken at 200 rpm for 15 min. The absorbances at the wavelength of 570 nm were measured using a microplate reader. The percentages of cell viability were calculated according to the following formula:

$$\% \,{\text{Cell}}\,{\text{viability}} = \left[ {{\text{A}}_{{{\text{sample}}}} /{\text{ A}}_{{{\text{control}}}} } \right] \times {1}00$$

where A_control_ is the absorbance of the control and A_sample_ is the absorbance of the samples.

### In vitro anti-aging activities

#### Collagen biosynthesis on human skin fibroblasts by Sirius Red assay

The collagen biosynthesis in human skin fibroblasts induced by the samples was assayed using the Sirius Red method, as described previously^9,17^. A density of 5 × 10^5^ cells/well of human skin fibroblasts were seeded into a sterile 6-well plate and adjusted the volume to 1.8 mL with DMEM medium. The cells were incubated at the standard conditions for 24 h. After incubation, the cells were treated with 200 µL of the samples at the proper concentrations and then incubated at the standard conditions overnight. Vitamin C was used as the positive control. Subsequently, the medium was removed and the cells were gently washed with PBS for 3 times.

An amount of 1 mL of 0.1% (w/v) Sirius red (Sigma Aldrich-Merck KGaA, Darmstadt, Germany) solution in saturated picric acid (SRL, Maharashtra, India) was added to each well and further incubated at room temperature for 1 h. After the dye removal, the plates were washed with 1 mL of 10 mM HCl for 5 times, and dissolved by adding 1 mL of 0.1 M NaOH. The plates were gently shaken at 200 rpm for 15 min. The absorbances of the lysate at the wavelength of 540 nm were measured using a microplate reader. The percentages of collagen contents were calculated according to the following formula:

$$\% \,{\text{Collagen}}\,{\text{content}} = \left[ {{\text{C}}_{{{\text{sample}}}} /{\text{ C}}_{{{\text{control}}}} } \right] \, \times { 1}00$$

where C_control_ is the absorbance of the control, and C_sample_ is the absorbance of the samples.

#### Anti-melanogenesis on B_16_F_10_ cells

The anti-melanogenesis activity of the samples on B16F10 cells was assayed using the melanin content assay, as described previously^9,17^. A density of 2.5 × 10^5^ cells/well of B16F10 cells were seeded into a sterile 6-well plate and adjusted the volume to 1.8 mL with DMEM medium. The cells were incubated at the standard conditions for 24 h. After incubation, the cells were treated with 200 µL of the samples at the proper concentrations and then incubated at the standard conditions for 72 h. Kojic acid was used as the positive control. Subsequently, the supernatants were collected to clean microfuge tubes, whereas the cells were then washed with PBS for 3 times, dissolved in 200 µL of 10%(w/v) NaOH, and incubated at 60 °C for 1 h. The absorbances of the cell lysate at the wavelength of 450 nm were measured using a microplate reader. The percentages of the anti-melanogenesis were calculated according to the following formula:

$$\% \,{\text{Anti-melanogenesis}} = {1}00 - \left[ {\left( {{\text{M}}_{{{\text{sample}}}} /{\text{M}}_{{{\text{control}}}} } \right) \, \times { 1}00} \right]$$

where M_control_ is the absorbance of the control, and M_sample_ is the absorbance of the samples.

#### Expression of SIRT1 and FOXO1 mRNA on dermal human skin fibroblasts by qRT-PCR

The expression of SIRT1 and FOXO1 mRNA in dermal human skin fibroblasts was assayed using the quantitative reverse transcription polymerase chain reaction (qRT-PCR) method, as described previously^20^. A density of 5 × 10^5^ cells/well of dermal human skin fibroblasts were seeded into a sterile 6-well plate and adjusted the volume to 1.8 mL with DMEM medium. The cells were incubated at the standard conditions for 24 h. After incubation, the cells were treated with 200 µL of the samples at the proper concentrations and then incubated at the standard conditions for 24 h. Resveratrol (Sigma Aldrich-Merck KGaA, Darmstadt, Germany) was used as the positive control. Subsequently, the medium was removed and the cells were gently washed with PBS. The total RNA was isolated with NucleoSpin RNA Plus (Macherey Nagel, Germany) according to the manufacturer’s instructions and quantified by Qubit (Invitrogen, USA). The specific primers for the SIRT1, FOXO1, and β-actin genes were purchased from Macrogen, Singapore. The sequences of the specific primers were as follows: SIRT1: 5′-TAG CCT TGT CAG ATA AGG AAG GA-3′ (forward), 5′-ACA GCT TCA CAG TCA ACT TTG T-3′ (reverse); FOXO1: 5′-GAC GCC GTG CTA CTC GTT-3′ (forward) and 5′-CGG TTC ATA CCC GAG GTG-3′ (reverse); and β-actin: 5′TCA TGC AGT GTG ACG TTG ACA TCC GT-3′ (forward), 5′-CCT AGA AGC ATT TGC GGT GCA CGA TG -3′ (reverse). The qRT-PCR mixtures contained 1 × RNA-direct SYBR green Master mix (Toyobo, Japan), 5 mM Mg(OAc), 250 nM of the specific primer, and 1 µg of total RNA in the total volume of 20 µL. The qRT-PCR reactions were performed on Mx3005P (Agilent Technologies, USA) under the amplification condition following parameters: 90 °C for 30 s and 60 °C for 20 min for reverse transcription, 45 cycles at 95 °C for 15 s, 60 °C for 15 s, and 74 °C for 10 s. After qRT-PCR, the relative quantity of mRNA was determined by the 2 − ΔΔCT method (21) using β-actin as a reference gene.

### Statistical analysis

The independent experiments were performed in triplicate to ensure reproducibility. All data were presented as the mean ± standard deviation (SD). Statistical differences between groups were examined by analysis of variance (ANOVA) with the Tukey test at a significance level of *p* < 0.05.

## Results

### The extraction yields, characteristics and phytochemical constituents of the GIG extracts

The extraction yields, characteristics, and phytochemical constituents of the Giant Indian Gooseberry (GIG) extracts were shown in Table 1. The extraction yields of the GIG extracts ranged from 2.17% (the GIG-S-M extract) to 8.19% (the GIG-J-L extract). The main phytochemical constituents of all GIG extracts were glucosides, flavonoids, and tannins.

**Table 1:** The extraction yields, characteristics and phytochemical constituents of the Giant Indian goose-berry extracts.

Extracts	% Yields	Characteristics	Phytochemical Constituents						
			Alkaloids	Flavonoids	Glycosides	Saponin	Tannins	Terpenoids	Xanthones
GIG-J-L	8.19	Viscous, yellow–brown	−	++++	+++	+	++	+	−
GIG-R-B	7.79	Viscous, dark green–brown	−	++	++	+	+	−	−
GIG-R-M	3.17	Viscous, dark brown	+	++	+	−	++	+	+
GIG-S-B	6.06	Viscous, dark green	−	++	++	+	++	−	−
GIG-S-M	2.17	Viscous, dark brown	−	++++	+++	−	+++	+	−

The qualitative results are expressed as (+) for the presence and (−) for the absence of phytochemicals. + is low; ++ is moderate; ++ is strong; ++++ is very strong. GIG is the Giant Indian gooseberry extracts. J, S, R is the juice filtrate, seeds and meat residues, respectively. M is the Maceration process in 95% (v/v) ethanol. B is the Boiling extraction with distilled water. L is lyophilization by a freeze dryer.

### Total phenolic contents, total flavonoid contents and Quercetin content of the GIG extracts

**Figure 1:**
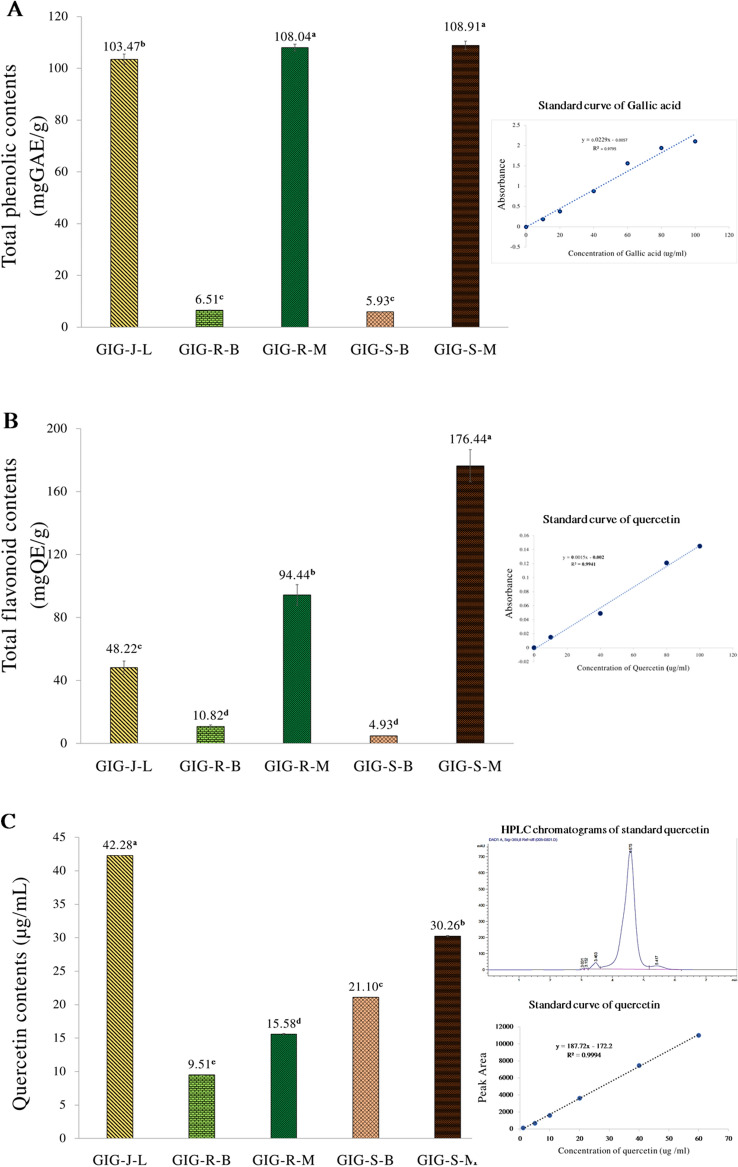
Total phenolic contents (**A**), Total flavonoid contents (**B**) and Quercetin contents (**C**) of the Giant Indian gooseberry extracts. The data are expressed as mean ± SD, and different superscript asterisks in the column indicate the significant differences at *p* < 0.05 by the Tukey test. GIG is the Giant Indian gooseberry extract. J, S, and R are the juice filtrate, seeds and meat residues, respectively. M is the Maceration process in 95% (v/v) ethanol. B is the Boiling extraction with distilled water. L is lyophilization by a freeze dryer.

The Folin-Ciocalteu and the aluminum chloride method are the most common for determining the total phenolic and flavonoid compounds. Figure 1 shows the total phenolic, flavonoid, and quercetin contents of the Giant Indian Gooseberry extracts. The GIG extracts from seeds by the maceration process (GIG-S-M) gave the highest total phenolic (108.91 ± 1.63 mgGAE/g) and flavonoid contents (176.44 ± 10.28 mgQE/g), whereas the GIG extracts from lyophilized juice (GIG-J-L) showed the highest quercetin contents by the HPLC assay of 42.28 ± 0. 30 µg/mL at the significant level of *p* < 0.05.

### Anti-oxidative activities of the GIG extracts

All of the GIG extracts exhibited the three anti-oxidative activities, including the free radical scavenging activity, the lipid peroxidation inhibition and the metal chelation (Table 2). The GIG-J-L, GIG-S-M, and GIG-R-M extracts gave the significant highest free radical scavenging activity by the DPPH assay that were comparable to standard vitamin C (SC_50_ of 0.05 ± 0.01 mg/mL) at the *p* value < 0.05. In addition, the GIG-R-B and GIG-S-B extracts showed the significant highest lipid peroxidation inhibition (LC_50_ of 2.38 ± 0.05 mg/mL), and the metal chelation activity (MC_50_ of 2.61 ± 0.12 mg/mL), but they were lower than that of the standard vitamin E (LC_50_ of 0.40 ± 0.61 mg/mL) and EDTA (MC_50_ of 0.95 ± 0.16 mg/mL), respectively (*p* < *0.05*).

**Table 2:** Anti-oxidative activities of the Giant Indian gooseberry extracts.

Extracts	Anti-oxidative activities		
	Free radical scavenging activity (SC_50_ mg/ml)	Lipid peroxidation inhibition (LC_50_ mg/ml)	Metal chelation (MC_50_ mg/ml)
GIG-J-L	0.05 ± 0.01^a^	3.36 ± 0.13^C^	8.91 ± 0.31^iv^
GIG-R-B	1.05 ± 0.12^c^	2.38 ± 0.05^B^	4.90 ± 0.75^iii^
GIG-R-M	0.05 ± 0.01^a^	4.66 ± 0.31^D^	6.68 ± 0.12^iii^
GIG-S-B	0.21 ± 0.01^b^	3.18 ± 0.03^C^	2.61 ± 0.12^ii^
GIG-S-M	0.05 ± 0.01^a^	3.44 ± 0.38^C^	13.91 ± 0.70^v^
Std. vitamin C	0.05 ± 0.05^a^	–	–
Std. vitamin E	–	0.95 ± 0.16^A^	–
Std. EDTA	–	–	0.40 ± 0.61^i^

The data are expressed as mean ± SD and different superscript asterisks (^a–c^ for SC_50_, ^A–D^ for LC_50_, and ^i–v^ for MC_50_) in the column indicate the significant differences at *p* < 0.05 by Tukey test. GIG is the Giant Indian gooseberry extracts. J, S, R is the juice filtrate, seeds, and meat residues, respectively. M is the Maceration process in 95% (v/v) ethanol. B is the Boiling extraction with distilled water. L is lyophilization by a freeze dryer.

### Mushroom tyrosinase inhibition activities of the GIG extracts

**Figure 2:**
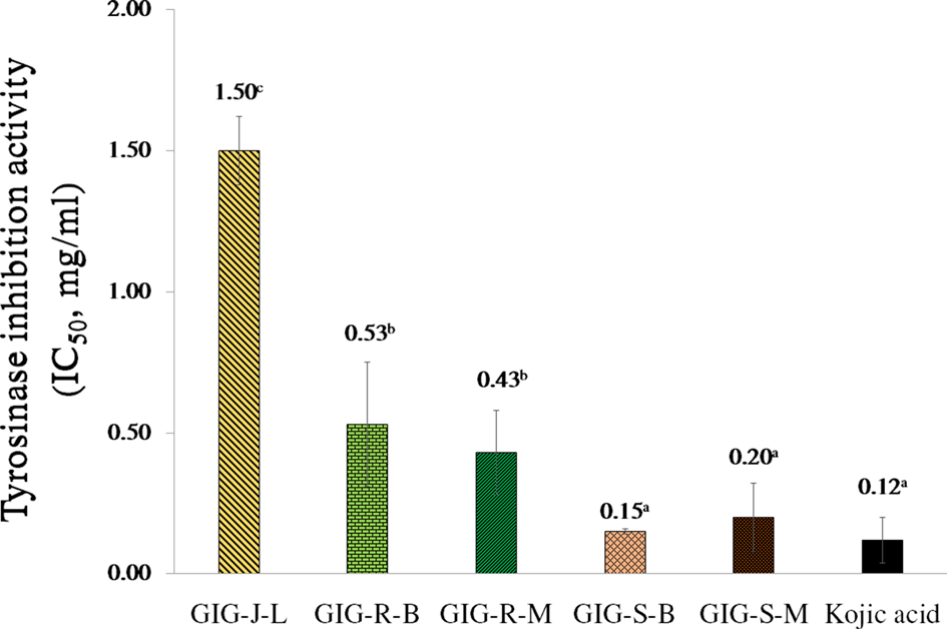
Mushroom tyrosinase inhibition activities (IC_50_ mg/ml) of the Giant Indian gooseberry extracts. The data are expressed as mean ± SD, and different superscript letters (^a–c^) in the column indicate the significant differences at *p* < 0.05 by the Tukey test. GIG is the Giant Indian gooseberry extract. J, S and R are the juice filtrate, seeds and meat residues, respectively. M is the Maceration process in 95% (v/v) ethanol. B is the Boiling extraction with distilled water. L is lyophilization by a freeze-dryer.

All of the GIG extracts exhibited the mushroom tyrosinase inhibition activity (Fig. 2). The GIG extract from the seeds extracted by the Maceration method (GIG-S-M) and the Boiling method (GIG-S-B) gave the highest inhibition activity, which was comparable to the standard kojic acid (IC_50_ of 0.12 ± 0.08 mg/mL) at the significant of *p* value < 0.05.

### Cytotoxicity of the GIG extracts

**Figure 3:**
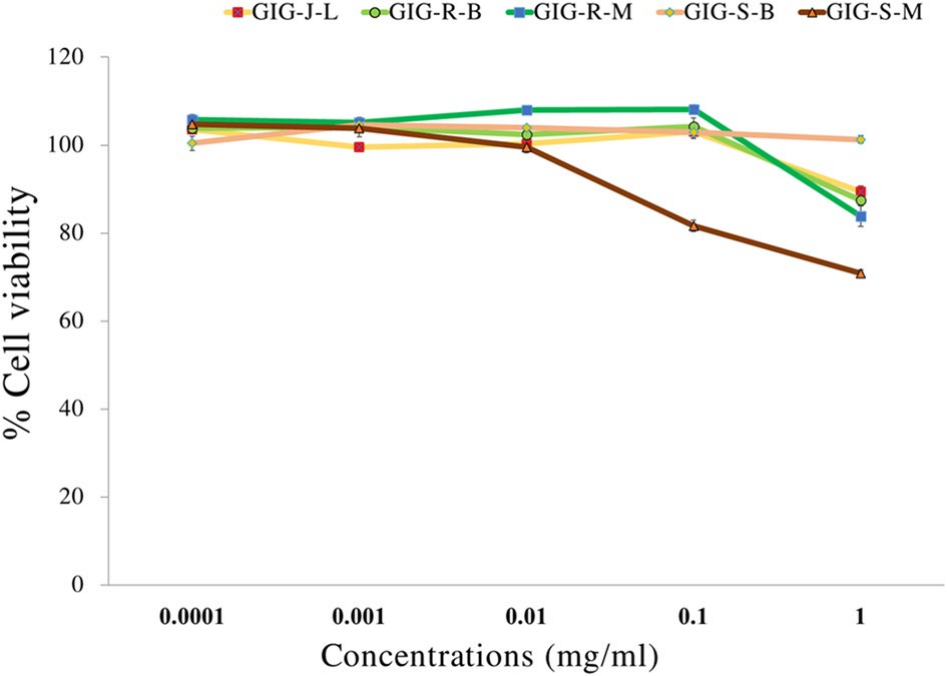
Cytotoxicity of the Giant Indian gooseberry extracts at various concentrations on the human skin fibroblasts. The data are expressed as mean ± SD. GIG is the Giant Indian gooseberry extract. J, S, and R are the juice filtrate, seeds and meat residues, respectively. M is the Maceration process in 95% (v/v) ethanol. B is the Boiling extraction with distilled water. L is lyophilization by a freeze-dryer.

Figure 3 demonstrated the cytotoxicity on human skin fibroblasts by the MTT assay of the GIG extracts. All GIG extracts at the concentrations of 0.1 mg/mL gave more than 80% relative cell viability when compared to the control (the un-treated), resulting to have no any cytotoxicity on the human skin fibroblasts^10^. However, the higher concentration (1 mg/mL) of all of the GIG extracts would be considered cytotoxic for the cells. The higher concentrations than 1 mg/mL did not test since they could not completely dissolve in the solvent.

### Stimulation of collagen biosynthesis of the GIG extracts

**Figure 4:**
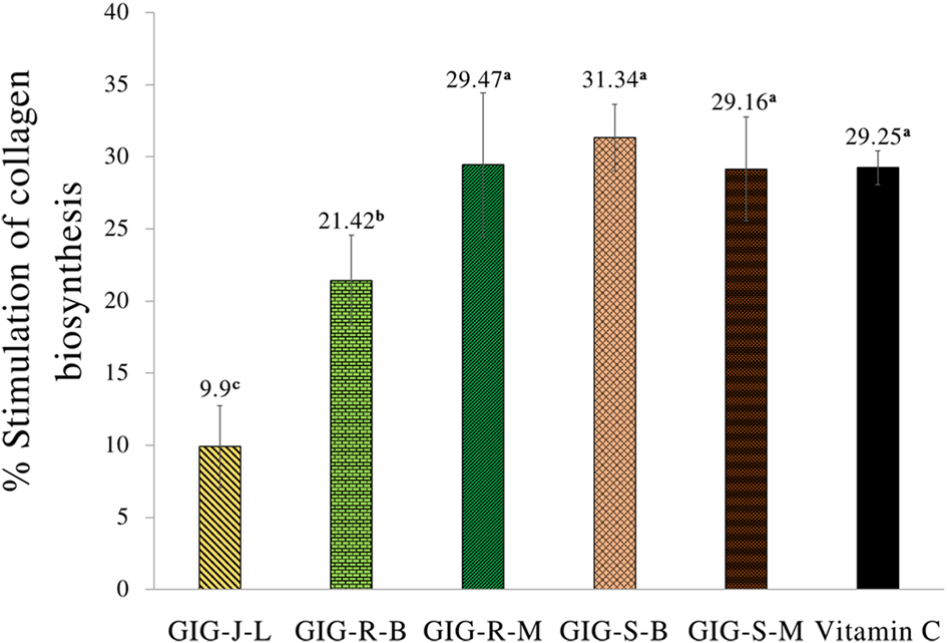
Stimulation of collagen biosynthesis on the human skin fibroblasts. The data are expressed as mean ± SD, and different superscript letters (^a–c^) in the column indicate the significant differences at *p* < 0.05 by the Tukey test. GIG is the Giant Indian gooseberry extract. J, S, and R are the juice filtrate, seeds and meat residues, respectively. M is the Maceration process in 95% (v/v) ethanol. B is the Boiling extraction with distilled water. L is lyophilization by a freeze-dryer.

Following the cytotoxicity on the human skin fibroblasts of the GIG extracts above, the proper concentration of the GIG extracts for investigating the stimulation of collagen biosynthesis was 0.1 mg/mL. All of the GIG extracts showed the stimulation of collagen biosynthesis on the human skin fibroblasts determined by the Sirius Red method (Fig. 4). The GIG-S-B, GIG-S-M, and GIG-R-M extracts at the concentrations of 0.1 mg/mL exhibited the significant highest stimulation of collagen biosynthesis of 31.34 ± 2.31%, 29.16 ± 3.60% and 29.47 ± 4.97%, respectively, which were comparable to the standard vitamin C at 29.25 ± 1.18% (*p* < *0.05*).

### Anti-melanogenesis of the GIG extracts

**Figure 5:**
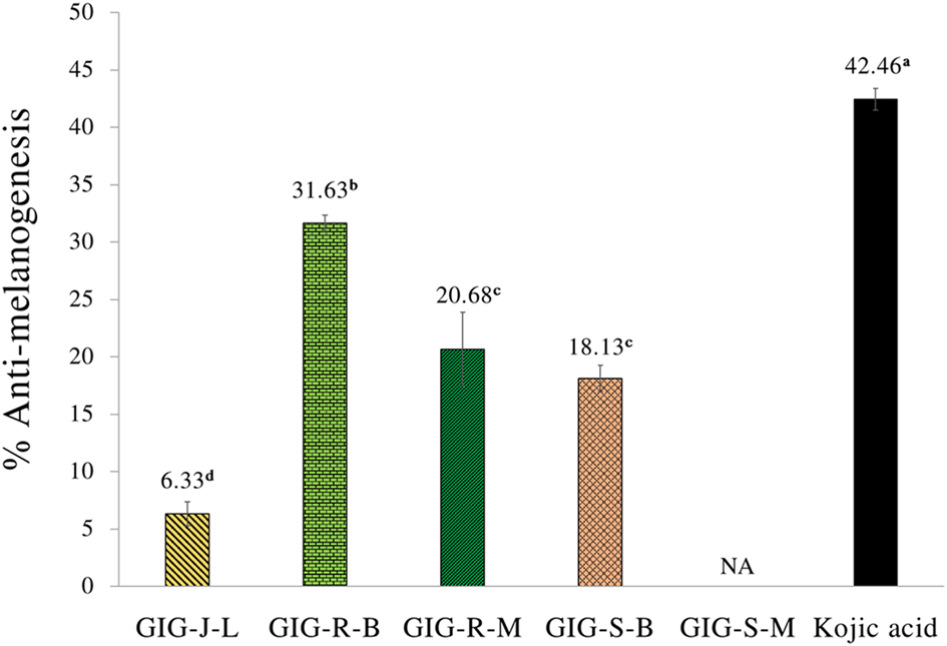
Anti-melanogenesis on murine melanomas (B_16_F_10_) cell lines of the Giant Indian gooseberry extracts at a concentration of 0.1 mg/ml. The data are expressed as mean ± SD, and different superscript letters (^a–d^) in the column indicate the significant differences at *p* < 0.05 by the Tukey test. GIG is the Giant Indian gooseberry extract. J, S, and R is the juice filtrate, seeds and meat residues, respectively. M is the Maceration process in 95% (v/v) ethanol. B is the Boiling extraction with distilled water. L is lyophilization by a freeze-dryer.

Figure 5 revealed the anti-melanogenesis on the murine melanomas (B_16_F_10_) cells of the GIG extracts at the concentration of 0.1 mg/mL. This concentration of all the GIG extracts showed no cytotoxicity on the B_16_F_10_ cells (data not shown). The GIG extract from the meat residues extracted by the Boiling extraction (GIG-R-B) exhibited the significant highest anti-melanogenesis of 31.63 ± 0.70%, but it was lower than that of the standard kojic acid (42.46 ± 0.94%) at the *p* value < 0.05.

### Activation of anti-aging genes expression of the GIG extracts

**Figure 6:**
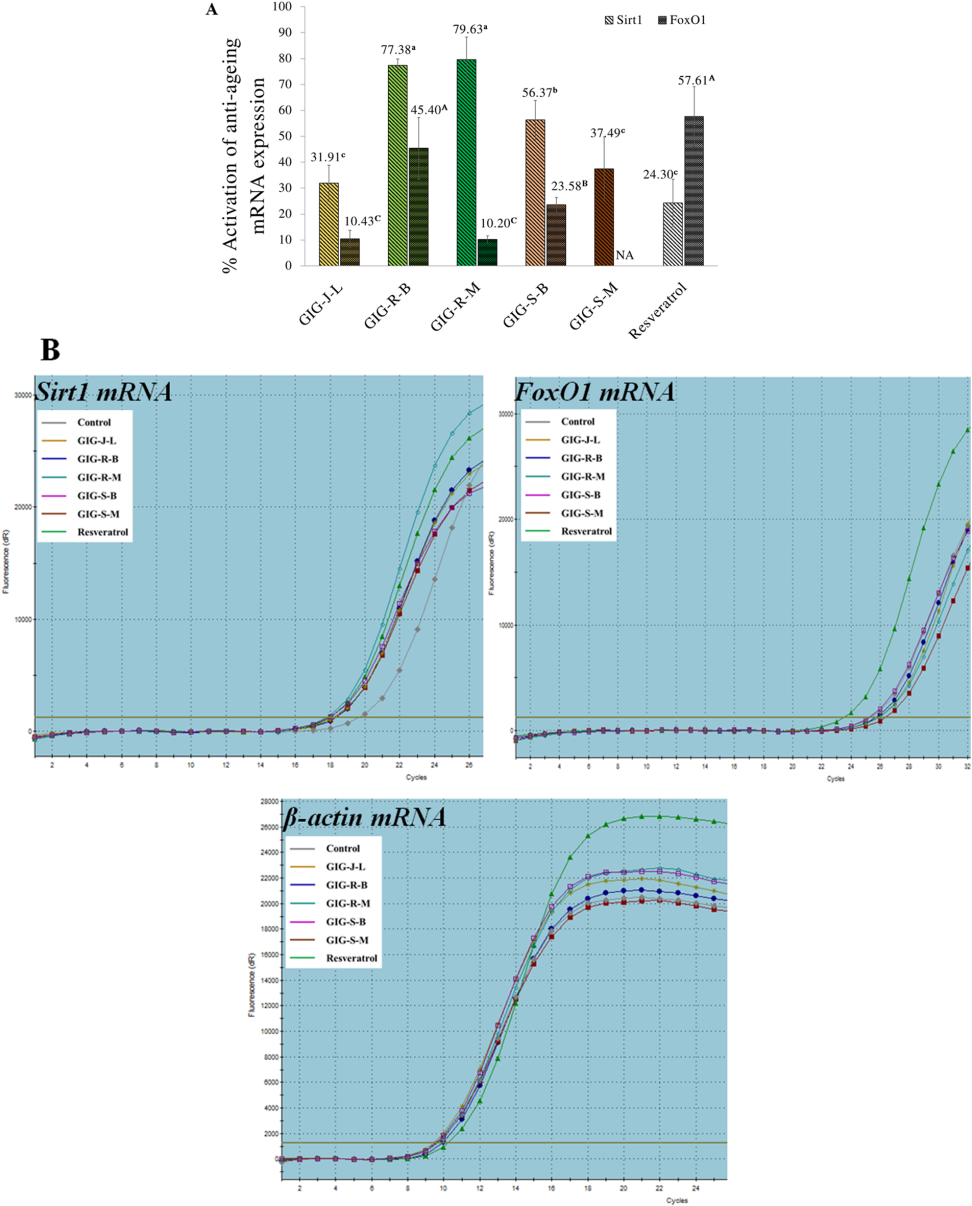
Activation of anti-aging genes on human skin fibroblasts (**A**) and amplification plots of gene expression (**B**) of the Giant Indian gooseberry extracts. The data are expressed as mean ± SD and different superscript letters (^a–c^ for *SIRT1* and ^A-C^ for *FOXO1*) in the column, indicating the significant differences at *p* < 0.05 by the Tukey test. NA is no activity. GIG is the Giant Indian gooseberry extract. J, S, and R are the juice filtrate, seeds and meat residues, respectively. M is the Maceration process in 95% (v/v) ethanol. B is the Boiling extraction with distilled water. L is lyophilization by a freeze-dryer.

The expression of anti-aging genes including *SIRT1* and *FOXO1* genes on the human skin fibroblasts of the GIG extracts was determined by the qRT-PCR technique. The *SIRT1* and *FOXO1* complementary DNA products were presented at sizes of 160 and 495 bp, respectively. Following the Fig. 6, the GIG extract from the meat residues extracted by the Boiling extraction (GIG-R-B) at the concentration of 0.1 mg/mL gave the significant highest activation both of *SIRT1* and *FOXO1* mRNA expression of 77.38 ± 2.52% and 45.40 ± 11.90%, respectively. Interestingly, all of the GIG extracts demonstrated the superior activation activity on *SIRT1* mRNA expression than that of the standard resveratrol (24.30 ± 9.10%) (*p* < *0.05*). In addition, the activation of *SIRT1* mRNA expression of the GIG-R-B extract were significantly dramatically superior to the standard resveratrol of 3 folds, whereas comparable to the standard resveratrol for the activation of *FOXO1* mRNA expression. Although, almost of the GIG extracts demonstrated the activation of anti-aging gene expression, but only the GIG-S-M extracts did not activate the *FOXO1* mRNA expression.

## Discussion

The plant genus Phyllanthus has been utilized as alternative and traditional herbal formulations for many centuries in Brazil, India, China and Southeast Asian countries^22^. Although both species of *P. indofischeri* Bennet (Giant Indian Gooseberry) and *P. emblica* L. have been used in Thailand for the treatments of diarrhea, leukorrhagia, cough, parasitosis, gastrointestinal chronic diseases, and for hair and skincare^16,22^, there is a limited amount of research on the Giant Indian Gooseberry (GIG) on its biological and pharmaceutical activities, especially for dermatological health and aesthetic purposes. In this study, the fruit meat and seeds of GIG were collected from Sra Kaeo Province, an eastern part of Thailand, and were extracted by the different methods, including the juice squeezing, the Maceration process and the Boiling extraction, respectively. Although several scientists reported that the Phyllanthus species, especially *P. emblica* L. present tannins, flavonoids, alkaloids, terpenoids, phenolic compounds, saponins, and glycosides as their phytochemicals^22^, there is no report for phytochemicals in *P. indofischeri* Bennet. To assess the in vitro anti-aging efficiency of the GIG extracts, is not only necessary to gain a new knowledge of the other Phyllanthus plant species, but also beneficial to be further developed as an active ingredient for dermatological and aesthetic uses from the local plants of Sra Kaeo Province as well.

From our finding, it revealed that the GIG extracts contain glycosides, flavonoids, and tannins as the main phytochemicals, and also present the total flavonoid (TFC) and phenolic (TPC) compounds as well as the quercetin level, which is one of the main bioactive compounds in Phyllanthus plants. However, the extraction yields (%) of the GIG extracts by the boiling extraction seemed to be higher than those from the Maceration process, but there are absences of heat-labile compounds such as alkaloids, steroids, xanthones, carotenoids, and terpenoids. The differences in extraction yields, phytochemicals, TPC, TFC, and quercetin of the GIG extracts might be affected by the solvents, extraction processes, and temperatures^10^. HPLC is a technique that is used to separate and identify single compounds based on their molecular properties. It can be used to measure the concentration of quercetin in a sample with high accuracy. In contrast, phenols and flavonoids are a group of compounds that contain a hydroxyl group (-OH) attached to an aromatic ring^10^. However, they are not as easy to measure as quercetin using HPLC. The Folin-Ciocalteu and the aluminum chloride method are the most common for determining the total phenolic and flavonoid compounds in several researches^9–18^. According to several studies, quercetin, phenolic, and flavonoid compounds have many biological and pharmaceutical activities related to skin aging, including anti-oxidation, anti-inflammation, anti-bacterial, anti-viral, anti-psoriasis, wound healing, anti-itching, skin whitening, and photoprotection^23^. Therefore, the phenolic, flavonoid, and quercetin contents in the GIG extracts might be responsible for the anti-aging activities in this study (Supplementary Information).

Skin is exposed to air pollutants, including ultraviolet rays, diesel exhaust fumes, xenobiotics, etc., which can promote ROS production^24^. The radical oxygen species (ROS) cause age-associated damage at the cellular and tissue levels, which accelerates dermatological and aesthetic problems characterized by wrinkles and atypical pigmentation^24,25^. The three anti-oxidative model systems, which include free radical scavenging, lipid peroxidation inhibition, and metal chelation, have been widely used to investigate the anti-oxidative activities of various natural sources that can reduce skin diseases and aging^25^. For the free radical scavenging model, DPPH odd electrons are reduced by receiving a hydrogen atom from an anti-oxidant to be hydrazine molecule^26^, whereas the lipid peroxidation is the model that can inhibit the oxidation of copper (II) to copper (III) ion passed by linoleic acid^27^. For the metal chelation is based on the oxidation of copper (II) to form a ferric-ferrozine complex^28^. Anti-oxidative activities of the GIG extracts were presented with the SC_50_, LC_50_ and MC_50_ values for free radical scavenging activity, the lipid peroxidation inhibition, and metal chelation activity, respectively. In this study, all GIG extracts demonstrated the three anti-oxidative activities, possibly due to their presences of many phytochemicals such as glycosides, flavonoids, and tannins and especially quercetin. This is consistent with previous findings that *P. indofischeri* Bennett leaves and bark extracts prepared with water and ethanol have significant α-amylase inhibitory and antioxidant activity^29^. Khan et al.^30^ reported that the *P. emblica* L. fruit extracts, which contain phenolic and flavonoid compounds, showed the free radical scavenging activity, lipid peroxidation and reducing power activity. In comparison to my previous study, the GIG-S-M extract showed higher free radical scavenging and lipid peroxidation activities, but lower metal chelating activity than the pink rambutan seed extract prepared by maceration in 95% ethanol^31^. Quercetin contents in the GIG extracts might also be responsible for the three anti-oxidative activities since they have the potential to prevent many oxidation reactions that relate to age-related dermatological ailments^23,32^.

Tyrosinase is a copper-containing enzyme regarded as the rate-limiting enzyme that serves the hydroxylation of L-tyrosine to dihydroxyphenylalanine (L-dopa) and the oxidation of L-dopa to dopaquinone, followed by the control of melanin-type proteins such as human tyrosinase related protein-1 (TRP-1) and human tyrosinase related protein-2 (TRP-2) in melanogenesis on melanocytes^33^. Melanin overproduction can cause dark spots, dullness, melasmas, senile lentigo, and freckles, which are hallmarks of skin aging^33^. All GIG extracts exhibited the highest dose-dependent levels of mushroom tyrosinase inhibition and also anti-melanogenesis on B_16_F_10_ cells. These results are similar to the extract from Pink rambutan seeds from our the previous report^31^. The quercetin content of the GIG extracts may be responsible for inhibiting mushroom tyrosinase activity by binding to the active site of tyrosinase by hydrophobic interaction and chelating to copper ions with the 3′, 4′-dihydroxy groups, resulting in inhibition of the catalytic activity^34^. Quercetin can also down-regulate melanogenesis by α-MSH-stimulated microphthalmia-associated transcription factor (MITF), and tyrosinase related protein-1&2 (TRP1&2) in melanogenesis^35^. However, the GIG-S-M extract did not show anti-melanogenesis on B16F10 cells. Nonetheless, quercetin faces challenges in traversing the cell membrane of B16F10 cells efficiently because of poor solubility and bioavailability^36^. This obstacle arises due to the elevated expression of multidrug resistance protein 1 (MDR1) in these cells. MDR1 is a protein responsible for expelling various foreign molecules^37^, it might include quercetin.

Collagen is the primary structural component of the dermis and the most abundant protein and extracellular matrix found, and it is responsible for the strength and support of human skin. The breakdown of collagen in the skin resulting in a loss of durability and elasticity is the cutaneous sign of aging, which can be characterized by thickened fibrils organized in rope-like bundles in comparison to younger skin^38^. According to collagen biosynthesis, there is a complex cellular process that starts with transcription of collagen genes (COLs), followed by translation and translocation of the nascent polypeptide chain to the rough ER (rER), co-translational modification and folding, trafficking across the Golgi network, secretion; and extracellular processing and maturation^39^. Several anti-oxidants such as vitamins C, pycnogenol, co-enzyme Q10, green tea, idebenone, silymarin, and ferulic acid are incorporated into topical skincare products to promote the production of collagen. According to our results, it was revealed that all GIG extracts can stimulate the collagen biosynthesis on human skin fibroblasts as determined by the Sirius Red assay. Although there has been no previous research on the collagen biosynthesis of the extracts from *P. indofischeri* Bennet, some researches on *P. emblica* L. has been conducted on this stimulation activity. Chanvorachote et al*.*^40^ reported that the *P. emblica* extract at a concentration of 0.1 mg/mL significantly increased the type I pro-collagen level to 1.65–6.78 folds greater than that of the untreated, and also inhibited collagenase activity in a dose-dependent manner. Stipcevic et al.^41^ described that quercetin has the ability to directly influence the collagen synthesis and can inhibit the collagenolytic enzymes, precisely matrix metalloproteinases (MMPs), by decreasing free zinc-ion concentrations caused by chelation agents, resulting in an increase in the rate and amount of synthesized collagen by fibroblasts. Thus, quercetin might be responsible for the stimulation of collagen biosynthesis in the human skin fibroblasts.

Sirtuin 1 (SIRT1) regulates chromatin silencing and transcriptional repression in dependence on the energetic state of the cell^41^. There is evidence that they play a role in oxidative stress response, glucose metabolism, mitochondrial function, cell differentiation, neuroprotection, insulin secretion, vascular protection, and thus the development of several age-related diseases^7,42^. SIRT1 has a wide range of target substrates, including a transcriptional factor forkhead box class O1 (FOXO) 1 which is deacetylated at three lysine residues within the forkhead DNA binding domain by SIRT1^43^. Forkhead transcription factor O1 (FOXO1) plays a role in multiple biological processes including oxidative stress, apoptosis, and cell cycle arrest^6^. Previous research has shown that SIRT1 and FOXO1 can delay aging mechanisms in oxidative phosphorylation system while restoring mitochondrial dysfunction in species ranging from invertebrates to mammal such as the development of aging, oxidative stress resistance, insulin resistance, and metabolism, including skin aging^6,44^.

Currently, Up-regulations of the *SIRT1* and *FOXO1* mRNA expressions on various cells of natural plant extracts have been reported in vitro and in vivo. The *Prunus mume* seed extract can increase the level of collagen and the *SIRT1* mRNA expression and decreases the metalloproteinase 1 mRNA expression on mouse back dorsal skin tissue^5^. The extract from *Dracocephalum kotschyi* significantly up-regulates p-AKT, p-FOXO1, PPAR, and SREBP-1 expressions in adipose tissue^45^. The extracts of *Retama monosperma* (L.) Boiss. seed and flower, and its bioactives, including quercetin, genistein, kaempferol, and 6-methoxykaempferol, can stimulate *SIRT1* gene expression in HaCaT cells^46^. However, there is no report in the literature of Phyllanthus plants on *SIRT1* and *FOXO1* genes. In our study, the activation of *SIRT1* and *FOXO1* mRNA expression by the extracts from *P. indofischeri* Bennet has been reported for the first time. The results demonstrated that all the GIG extracts can up-regulate the levels of *SIRT1* and *FOXO1* mRNA expression on human skin fibroblasts. These stimulation effects are similar to resveratrol derived from grape skin, which is an effective SIRT1 and FOXO1 activator^47^. Several natural compounds abundantly found in vegetables and fruits that contain polyphenols (resveratrol, quercetin, curcumin, fisetin, apigenin, and luteolin) and non-polyphenols (berberine) have the potential to up-regulate *SIRT1* and *FOXO1* mRNA expression and activity, and to be considered for the prevention and treatment of stress-related oxidative diseases^12,13,47^. In comparison to my previous study, the GIG-S-M extract showed higher activation of *SIRT1* mRNA, activation of *FOXO1* mRNA than the pink rambutan seed extract prepared by maceration in 95% ethanol^31^. Studies have shown that flavonoids including quercetin can regulate a wide range of pathways by targeting the activity of SIRT1. These pathways include SIRT1/AMPK/NF-κB, SIRT1/Keap1/Nrf2/HO-1, and SIRT1/PI3K/Akt^48^. These pathways increase the activity of antioxidant enzymes and anti-inflammatory cytokines, as well as the efficiency of mitochondrial processes^48^. Therefore, it seems that the quercetin contents and their phytochemicals in the extracts from *P. indofischeri* Bennet might be responsible for the stimulation of *SIRT1* and *FOXO1* mRNA expression. Nevertheless, the GIG-S-M exhibited no signs of FOXO1 mRNA expression activation. This phenomenon could potentially be attributed to specific phytochemical components within the extract, notably tannins, a class of polyphenols known to interact with FOXO1, thereby impeding its activity and resulting in reduced FOXO1 mRNA expression^49^.

To assess the safety of natural active ingredients, all GIG extracts at the concentrations of 0.1 mg/mL showed no cytotoxicity on the human skin fibroblasts normal cells leading to an indication of no toxicity for dermal applications. The cytotoxic effect of the higher concentrations of the extracts observed during the experiments probably caused cell damage. The different cytotoxic effects of natural extracts depend on the concentration, time of exposure, and composition of extracts, as well as the types of cells that can respond to a specific plant extract^50^.

In conclusions, Giant Indian Gooseberry (*P. indofischeri* Bennet) has been mostly cultivated in Sra Kaeo Province, in the eastern part of Thailand, for use as food and Thai traditional medicine. Our findings show that the extracts from Giant Indian Gooseberry (GIG) have a high efficiency for in vitro anti-aging activity. We highlight the Giant Indian Gooseberry extracts from the meat residues and the seeds extracted by boiling (the GIG-R-B and GIG-S-B extracts) as an anti-wrinkle and whitening agent because they exhibited the highest activation of the anti-aging *SIRT1* and *FOXO1* genes and collagen biosynthesis, as well as tyrosinase inhibition and anti-melanogenesis, respectively. The outcome of this research can be translated into relevant applications in skin care in the future by developing new skin care products that contain Giant Indian Gooseberry (GIG) extracts because they have potent anti-aging activity and are non-cytotoxic to normal human skin cells. According to the findings of this study, giant Indian gooseberry extracts are a promising alternative source of natural anti-skin aging agents that can be further developed for dermatological health and wellness applications such as cosmetics, cosmeceuticals, and pharmaceuticals. Further studies are required to fraction or isolate anti-aging compounds from the giant Indian gooseberry extracts as well as the improvement of skin permeation and efficiency by nanotechnology. This would be got more higher efficiency and safety from the GIG extracts as an anti-aging natural source.

### Supplementary Information

Supplementary Information.

## Data Availability

All data generated or analysed during this study are included in this published article [and its supplementary information files].
