# Supplementary material for: Assessment of in vitro anti-skin aging activities of Phyllanthus indofischeri Bennet extracts for dermatological and aesthetic applications
Source: Sci Rep. 2023 Oct 31;13:18661. doi: 10.1038/s41598-023-45434-3 (PMC10618208; doi:10.1038/s41598-023-45434-3)
Supplement: Supplementary file 1 — Supplementary Information. [file 41598_2023_45434_MOESM1_ESM.docx]

**Supporting Information**

**Assessment of *in vitro* anti-skin ageing activities of *Phyllanthus indofischeri* Bennet extracts for dermatological and aesthetic applications**

**Korawinwich Boonpisuttinant^1^, Thanachai Taka^2^, Warintorn Ruksiriwanich^3^, Romchat Chutoprapat^4^, Sarinporn Udompong^1^, Rattiya Kansawang^1^, Jinapa Sangsee^1^, Wirinda Chompoo^1^, Kitrawi Samothai^1^, and Ratakorn Srisuttee^5^***

*^1^Innovative Natural Products from Thai Wisdom Research Unit, Faculty of Integrative Medicine, Rajamangala University of Technology Thanyaburi, Pathumthani 12130, Thailand*

*^2^iCell Research Institute Laboratory Unit, Bangkok 10230, Thailand*

*^3^Faculty of Pharmacy, Chiang Mai University, Chiang Mai 50000, Thailand*

*^4^Department of Pharmaceutics and Industrial Pharmacy, Faculty of Pharmaceutical Sciences, Chulalongkorn University, Bangkok 10330, Thailand*

*^5^Faculty of Medicine, King Mongkut's Institute of Technology Ladkrabang, Bangkok 10520, Thailand*

Contact information: [korawinwich_b@rmutt.ac.th](mailto:korawinwich_b@rmutt.ac.th) (KB); E-mail: [ratakorn.sr@kmitl.ac.th](mailto:ratakorn.sr@kmitl.ac.th) (RS)

**Table S1.** The calculation of *SIRT1* mRNA expression

| **#1** | **SIRT1 mRNA** | | | | | | |
| --- | --- | --- | --- | --- | --- | --- | --- |
|  | **Ct of SIRT1** | **Ct of actin** | **ΔCt** | **ΔΔCt** | **2^^-ΔΔCt^** | **% Expression** | **%Stimulation** |
| control | 18.74 | 21.46 | -2.72 | 0.00 | 1.00 | 100.00 | 0.00 |
| GIG-J-L | 18.80 | 22.03 | -3.23 | -0.51 | 1.42 | 142.41 | 42.41 |
| GIG-R-B | 18.44 | 21.99 | -3.55 | -0.83 | 1.78 | 177.77 | 77.77 |
| GIG-R-M | 18.57 | 22.22 | -3.65 | -0.93 | 1.91 | 190.53 | 90.53 |
| GIG-S-B | 18.71 | 23.18 | -4.47 | -1.75 | 3.36 | 336.36 | 236.36 |
| GIG-S-M | 18.41 | 21.64 | -3.23 | -0.51 | 1.42 | 142.41 | 42.41 |
| Resveratrol | 18.94 | 21.99 | -3.05 | -0.33 | 1.26 | 125.70 | 25.70 |
| **#2** | **SIRT1 mRNA** | | | | | | |
|  | **Ct of SIRT1** | **Ct of actin** | **ΔCt** | **ΔΔCt** | **2^^-ΔΔCt^** | **% Expression** | **%Stimulation** |
| control | 18.96 | 21.59 | -2.63 | 0.00 | 1.00 | 100.00 | 0.00 |
| GIG-J-L | 19.06 | 22.06 | -3.00 | -0.37 | 1.29 | 129.24 | 29.24 |
| GIG-R-B | 18.90 | 21.85 | -2.95 | -0.32 | 1.25 | 124.83 | 24.83 |
| GIG-R-M | 18.04 | 21.51 | -3.47 | -0.84 | 1.79 | 179.01 | 79.01 |
| GIG-S-B | 18.48 | 21.70 | -3.22 | -0.59 | 1.51 | 150.52 | 50.52 |
| GIG-S-M | 18.89 | 21.79 | -2.90 | -0.27 | 1.21 | 120.58 | 20.58 |
| Resveratrol | 19.18 | 22.01 | -2.83 | -0.20 | 1.15 | 114.87 | 14.87 |
| **#3** | **SIRT1 mRNA** | | | | | | |
|  | **Ct of SIRT1** | **Ct of actin** | **ΔCt** | **ΔΔCt** | **2^^-ΔΔCt^** | **% Expression** | **%Stimulation** |
| control | 19.60 | 10.40 | 9.20 | 0.00 | 1.00 | 100.00 | 0.00 |
| GIG-J-L | 19.56 | 10.77 | 8.79 | -0.41 | 1.33 | 132.87 | 32.87 |
| GIG-R-B | 19.05 | 10.65 | 8.40 | -0.80 | 1.74 | 174.11 | 74.11 |
| GIG-R-M | 19.58 | 10.60 | 8.98 | -0.22 | 1.16 | 116.47 | 16.47 |
| GIG-S-B | 19.57 | 10.97 | 8.60 | -0.60 | 1.52 | 151.57 | 51.57 |
| GIG-S-M | 19.00 | 10.81 | 8.19 | -1.01 | 2.01 | 201.39 | 101.39 |
| Resveratrol | 20.01 | 11.28 | 8.73 | -0.47 | 1.39 | 138.51 | 38.51 |
| **#4** | **SIRT1 mRNA** | | | | | | |
|  | **Ct of SIRT1** | **Ct of actin** | **ΔCt** | **ΔΔCt** | **2^^-ΔΔCt^** | **% Expression** | **%Stimulation** |
| control | 19.47 | 10.22 | 9.25 | 0.00 | 1.00 | 100.00 | 0.00 |
| GIG-J-L | 19.58 | 10.63 | 8.95 | -0.30 | 1.23 | 123.11 | 23.11 |
| GIG-R-B | 19.12 | 10.72 | 8.40 | -0.85 | 1.80 | 180.25 | 80.25 |
| GIG-R-M | 19.24 | 10.75 | 8.49 | -0.76 | 1.69 | 169.35 | 69.35 |
| GIG-S-B | 19.20 | 10.69 | 8.51 | -0.74 | 1.67 | 167.02 | 67.02 |
| GIG-S-M | 19.42 | 10.75 | 8.67 | -0.58 | 1.49 | 149.48 | 49.48 |
| Resveratrol | 20.94 | 11.93 | 9.01 | -0.24 | 1.18 | 118.10 | 18.10 |

**Table S2.** The calculation of *FoxO1* mRNA expression

| **#1** | **FoxO1 mRNA** | | | | | | |
| --- | --- | --- | --- | --- | --- | --- | --- |
|  | **Ct of FoxO1** | **Ct of actin** | **ΔCt** | **ΔΔCt** | **2^^-ΔΔCt^** | **% Expression** | **%Stimulation** |
| control | 24.2 | 21.46 | 2.74 | 0.00 | 1.00 | 100.00 | 0.00 |
| GIG-J-L | 24.56 | 22.03 | 2.53 | -0.21 | 1.16 | 115.67 | 15.67 |
| GIG-R-B | 24.12 | 21.99 | 2.13 | -0.61 | 1.53 | 152.63 | 52.63 |
| GIG-R-M | 24.85 | 22.22 | 2.63 | -0.11 | 1.08 | 107.92 | 7.92 |
| GIG-S-B | 25.6 | 23.18 | 2.42 | -0.32 | 1.25 | 124.83 | 24.83 |
| GIG-S-M | 25 | 21.64 | 3.36 | 0.62 | 0.65 | 65.07 | -34.93 |
| Resveratrol | 24.14 | 21.99 | 2.15 | -0.59 | 1.51 | 150.52 | 50.52 |
| **#2** | **FoxO1 mRNA** | | | | | | |
|  | **Ct of FoxO1** | **Ct of actin** | **ΔCt** | **ΔΔCt** | **2^^-ΔΔCt^** | **% Expression** | **%Stimulation** |
| GIG-J-L | 24.32 | 21.59 | 2.73 | 0.00 | 1.00 | 100.00 | 0.00 |
| GIG-R-B | 24.7 | 22.06 | 2.64 | -0.09 | 1.06 | 106.44 | 6.44 |
| GIG-R-M | 23.92 | 21.85 | 2.07 | -0.66 | 1.58 | 158.01 | 58.01 |
| GIG-S-B | 24.09 | 21.51 | 2.58 | -0.15 | 1.11 | 110.96 | 10.96 |
| GIG-S-M | 24.12 | 21.70 | 2.42 | -0.31 | 1.24 | 123.97 | 23.97 |
| Resveratrol | 24.54 | 21.79 | 2.75 | 0.02 | 0.99 | 98.62 | -1.38 |
| Res | 24.23 | 22.01 | 2.22 | -0.51 | 1.42 | 142.41 | 42.41 |
| **#3** | **FoxO1 mRNA** | | | | | | |
|  | **Ct of FoxO1** | **Ct of actin** | **ΔCt** | **ΔΔCt** | **2^^-ΔΔCt^** | **% Expression** | **%Stimulation** |
| control | 27.08 | 10.40 | 16.68 | 0.00 | 1.00 | 100.00 | 0.00 |
| GIG-J-L | 27.31 | 10.77 | 16.54 | -0.14 | 1.10 | 110.19 | 10.19 |
| GIG-R-B | 26.99 | 10.65 | 16.34 | -0.34 | 1.27 | 126.58 | 26.58 |
| GIG-R-M | 27.14 | 10.60 | 16.54 | -0.14 | 1.10 | 110.19 | 10.19 |
| GIG-S-B | 27.4 | 10.97 | 16.43 | -0.25 | 1.19 | 118.92 | 18.92 |
| GIG-S-M | 26.64 | 10.81 | 15.83 | -0.85 | 1.80 | 180.25 | 80.25 |
| Resveratrol | 27.2 | 11.28 | 15.92 | -0.76 | 1.69 | 169.35 | 69.35 |
| **#4** | **FoxO1 mRNA** | | | | | | |
|  | **Ct of FoxO1** | **Ct of actin** | **ΔCt** | **ΔΔCt** | **2^^-ΔΔCt^** | **% Expression** | **%Stimulation** |
| control | 26.72 | 10.22 | 16.50 | 0.00 | 1.00 | 100.00 | 0.00 |
| GIG-J-L | 27 | 10.63 | 16.37 | -0.13 | 1.09 | 109.43 | 9.43 |
| GIG-R-B | 26.69 | 10.72 | 15.97 | -0.53 | 1.44 | 144.39 | 44.39 |
| GIG-R-M | 27.09 | 10.75 | 16.34 | -0.16 | 1.12 | 111.73 | 11.73 |
| GIG-S-B | 26.85 | 10.69 | 16.16 | -0.34 | 1.27 | 126.58 | 26.58 |
| GIG-S-M | 26.82 | 10.75 | 16.07 | -0.43 | 1.35 | 134.72 | 34.72 |
| Resveratrol | 27.68 | 11.93 | 15.75 | -0.75 | 1.68 | 168.18 | 68.18 |


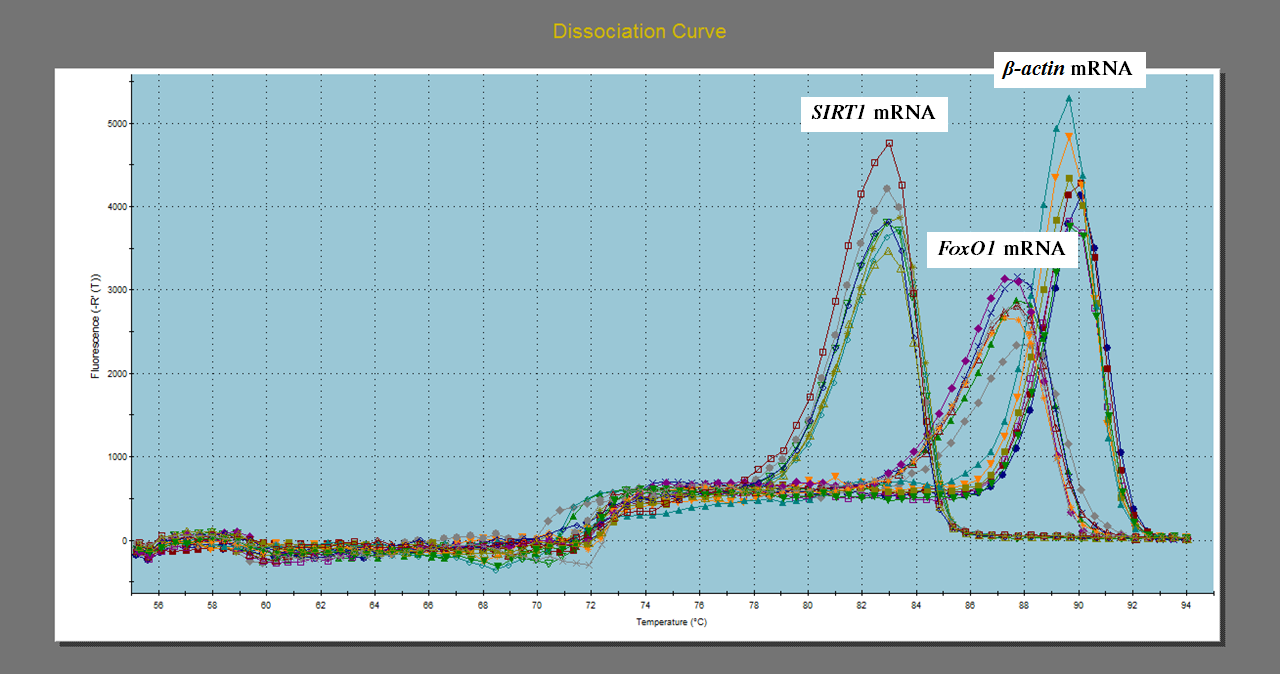


**Figure S1** Melting curve analysis of SYBR Green real-time PCR on the SIRT1, FoxO1 and β-actin genes.


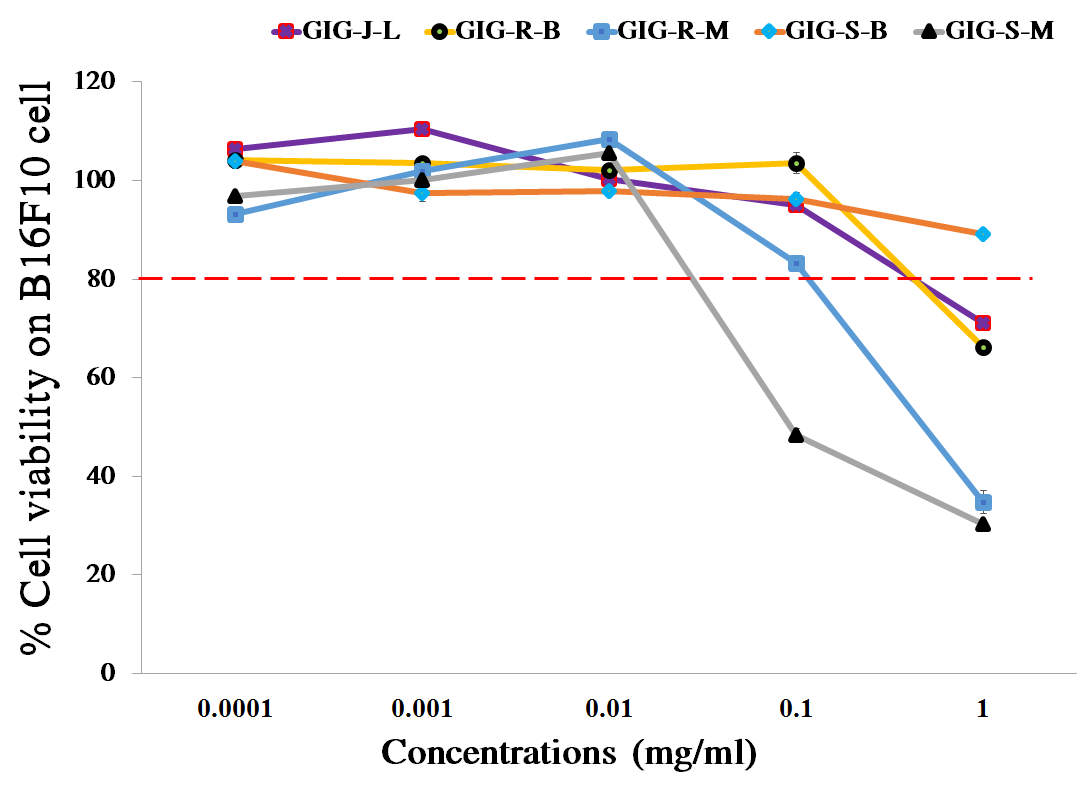


**Figure S2** Cytotoxicity of the Giant Indian gooseberry extracts at various concentrations on the B16F10 melanoma cells. The data are expressed as mean ± SD. GIG is the Giant Indian gooseberry extract. J, S, and R are the juice filtrate, seeds and meat residues, respectively. M is the Maceration process in 95% (v/v) ethanol. B is the Boiling extraction with distilled water. L is lyophilization by a freeze-dryer.
